# Immunohistochemical Expression of Programmed Death Ligand- 1 (PD-L1) in Colorectal Carcinoma; A Cross-sectional Study

**DOI:** 10.30699/IJP.2023.1988660.3054

**Published:** 2023-12-29

**Authors:** Shruti Tadachina, Sheela Devi Shivalingaiah, Mahesh Shetty

**Affiliations:** 1 Department of Pathology, JSS Medical College, JSS Academy of Higher Education & Research, Mysuru, Karnataka, India; 2 Department of Surgical Gastroenterology JSSMC & Hospital, Mysuru- 04, Karnataka, India

**Keywords:** Colorectal carcinoma, IHC, PD-L1 Expression, Tumor cells, Tumor-infiltrating immune cells

## Abstract

**Background & Objective:**

Colorectal carcinoma (CRC) is one of the most common cancers worldwide. The interaction of programmed cell death receptor 1 (PD-1) and programmed death ligand 1 (PD-L1) plays an important role by inhibiting the immune mechanism by which cancer cells escape antitumor immunity. Immunotherapy using checkpoint inhibitors is a growing treatment modality in many cancers; one such is anti-PD1/PD-L1. The present study aimed to study the immunohistochemical (IHC) expression of PD-L1 in CRC and its association with various known clinicopathological parameters.

**Methods:**

This study was a 2-year prospective study and included 34 colectomy specimens diagnosed as colorectal adenocarcinoma. The expression of PD-L1 was evaluated on tumoral cells and tumor-infiltrating immune cells (TIICs) and was correlated with various clinicopathological parameters.

**Results:**

Immunohistochemical expression of PD-L1 on tumoral cells and tumor microenvironment in CRC revealed positivity in 17.65% of cases each. The PD-L1 expression on tumoral cells was associated with lymphovascular invasion (LVI) and perineural invasion (PNI) with P- values of 0.012 and 0.005, respectively, while PD-L1 expression on TIICs was associated with tumor budding with a P-value of 0.022.

**Conclusion:**

IHC expression of PD-L1 on tumoral cells and immune cells may be associated with some known poor prognostic factors. Since anti-PD1/PD-L1 is used for targeted therapy, it may be beneficial and economically feasible to evaluate PD-L1 in CRC and establish its role as a prognostic factor.

## Introduction

Colorectal carcinoma (CRC) is the third most commonly diagnosed cancer worldwide, accounting for about 9.4% of all cancer-related fatalities (1). It is the fourth most prevalent cancer in men and the third most common cancer in women in India (2). 

Adenocarcinoma is the most frequent histological subtype of colorectal carcinoma, accounting for 90% of all cases (3). Cancer immunotherapy has a longer-lasting effect and is more tolerable than conventional therapies. Checkpoint blockade drugs targeting PD-1 and its ligand, PD-L1, have had exceptional clinical results in various cancers (4). The tumor cells showed expression of PD-L1 when tested by immunohistochemistry (IHC), in many tumors responding to therapy with anti-PD-1 (5). 

The objectives of this study were to evaluate the immunohistochemical expression of PD-L1 in colorectal carcinoma as well as its association with various clinicopathological parameters.

## Material and Methods

It was a prospective study of two years, from October 2018 to October 2020. Based on the the number of specimens received during the previous years in the department, a sample size was derived by the following formula. 

$S=\frac{\mathrm{Z2}\times P(1-p)}{\mathrm{d2}}=\frac{1.96\times 1.96\times 0.02\times 0.98}{0.05\mathrm{x0.05}}=$ 30

Where,

 Z= z-score (Confidence level = 95%), d= margin of error, p=prevalence.

A total of 34 colectomy specimens with histological diagnosis of adenocarcinoma in the Department of Pathology, JSS Hospital, Mysuru, were included in the study. The specimens were grossed as per the protocol. After documenting the gross features, representative tissue sections were taken. Routine histopathological processing was done, and sections were stained with hematoxylin and eosin (H&E) stain. The following variables were documented: age, gender, tumor location, tumor size, histological differentiation, LVI, PNI, and TNM (Tumor Node Metastasis) stage, according to the American Joint Committee on Cancer Staging Manual (AJCC) (6).

**Immunohistochemistry**

Immunohistochemical staining of PDL-1 was performed on formalin-fixed paraffin-embedded sections using commercially available antibodies (Biocare, Clone: CAL10) with Autostainer Intelipath from Biocare. The tonsil tissue was used as a positive control with moderate to strong staining intensity on lymphocytes and macrophages in germinal centers, with diffuse reticular crypt epithelial cells staining. 

**Scoring of PD-L1 Immunohistochemical Expression**

Membranous and cytoplasmic staining observed on tumor cells and /or tumor-infiltrating immune cells, which included lymphocytes, macrophages, dendritic cells, and histiocytes, was considered positive and scored. 

The intensity of staining of PD-L1 was scored as 0 for no staining, 1 for faint staining, 2 for moderate staining, and 3 for strong staining. >5% of the tumor cells and/or tumor-infiltrating immune cells showing PD-L1 expression with moderate or strong intensity were defined as positive (7). 

**Statistical Analysis**

The results were analyzed using SPSS 22 (SPSS Inc., Chicago, Ill., USA). Comparison between groups was performed with a Chi-square test (χ2) for categorical data. Associated P-values were reported for each of the baseline predictors. A P-value<0.05 was considered statistically significant.

## Results

**Clinicopathological Characteristics** (Table 1)

A total of 34 colectomy specimens were included in the study. The mean age of the patients was 57.5 years (range 30-85 years), with an M:F ratio of 1:1. The majority of the tumors were located in the left colon (55.88%), with ulceroproliferative growth as the predominant type (58.82%). The tumor size was ≥ 5 cm in 58.83% of the cases. The majority (91.17%) of the tumors were adenocarcinomas and were moderately differentiated. Lymphovascular and perineural invasion were seen in 64.7% and 17.65% of cases, respectively. The staging of most tumors was pT3 (61.76%) and N0 stage (41.17%). The pattern of invasion was infiltrative in 58.82% of cases. Intratumoral and peritumoral lymphocytic infiltration (TIL) was marked in 8.82% and 44.12% cases, respectively. Tumor budding was noted in 14.7% of cases, and mucin pools were reported in 38.24% of cases.

**Immunohistochemical expression of PD-L1**

**Fig. 1:**
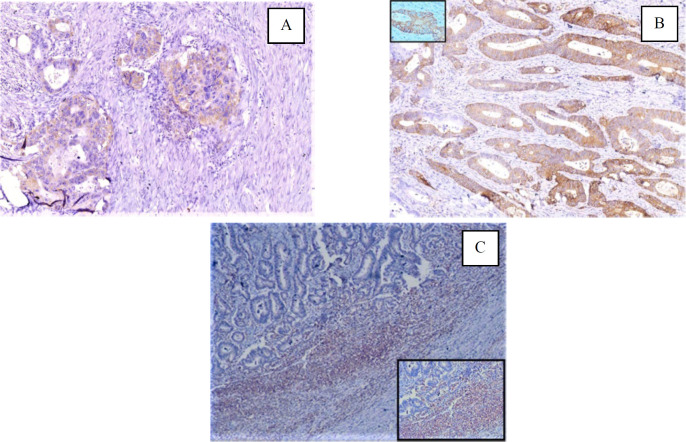
A) Cytoplasmic staining of PD-L1 in tumor cells (2+) (PD-L1, X200); (B) Membranous staining of PD-L1 in tumor cells (3+) (PD-L1, X100) [inset X400]; (C)PD-L1 staining of TIICs (2+), (PD-L1, X40) [inset X100]

**Fig. 2:**
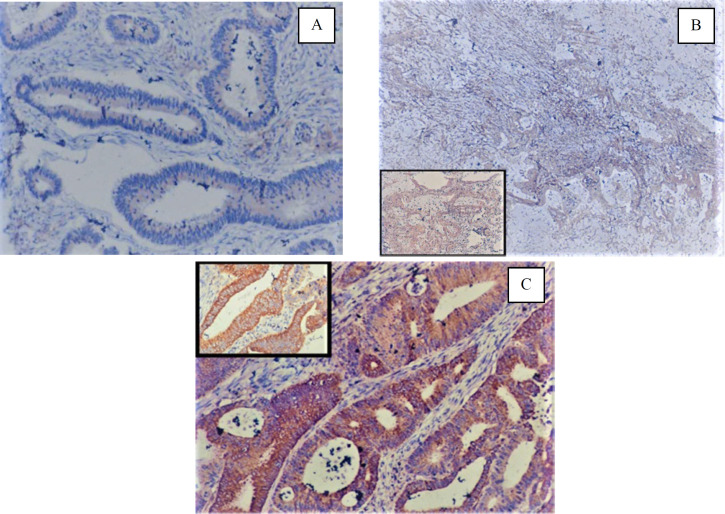
(A) PD-L1 cytoplasmic staining of tumor cells, (1+) (PD-L1, X100); (B) PD-L1 staining of tumor cells (2+) (PD-L1, X40) [inset X100]; (C) PD-L1 staining of tumor cells (3+) (PD-L1, X100) [inset X 400]

IHC expression of PD-L1 on tumor cells and tumor microenvironment in CRC was seen in 6 out of 34 cases (17.65%) each (Figures 1 & 2).

**Table 1:** Clinicopathological characteristics of CRC patients

**Parameter**	**Sample No.**
Mean Age	57.5
Gender	
**Male**	17
**Female**	17
Location	
Right	14
Left	19
Right & Left	1
Growth Type	
Ulceroinfiltrative	14
Ulceroproliferative	20
Tumor Size	
< 5cm	14
≥ 5cm	20
Differentiation	
Well	3
Moderate	31
Poor	0
Lymphovascular Invasion	
Yes	22
No	12
Perineural Invasion	
Yes	6
No	28
pT Staging	
T_1_	1
T_2_	10
T_3_	21
T_4_	2
pN Staging	
N_0_	14
N_1_	13
N_2_	7
Pattern of Invasion	
Pushing	14
Infiltrative	20
Intratumoral TILs	
None/Mild	31
Marked	3
Peritumoral TILs	
None/Mild	19
Marked	15
Tumor Budding	
Yes	5
No	29
Mucin Pool(s)	
Yes	13
No	21

**Relationship Between PD-L1 Expression on the Tumoral Cells, Tumor Infiltrating Immune Cells, and Clinicopathological Features**

IHC expression of PD-L1 on the tumoral cells was found statistically significant in association with LVI and PNI with a P-value of 0.012 and 0.005, respectively. In contrast, PD-L1 expression on the infiltrating immune cells is associated with tumor budding with a P-value of 0.022. However, no association was seen with other clinicopathological factors, including age, gender, location, histological type, grading, and staging (Tables 2 and 3).

**Table 2:** Immunohistochemical tissue expression of PD-L1 in the tumoral cells of the CRC patients

			PD-L1 (Tumor)			
			Negative		Positive	
Parameter			N (%)		N (%)	P-value
Age						0.551.
≥60 Years	13(86.7)			2(13.3)		
<60 years	15(78.9)			4(21.1)		
Gender						0.672
Male	14(82.4)			3(17.6)		
Female	14(82.4)			3(17.6)		
Location						0.102
Right	11(78.6)			3(21.4)		
Left	16(84.2)			3(15.8)		
Right & Left	1(100)			0(0)		
Growth Type						1.441
Ulceroinfiltrative	11(78.6)			3(21.4)		
Ulceroproliferative	17(85.0)			3(15.0)		
Tumor Size						5.346
< 5cm	9(64.3)			5(35.7)		
≥ 5cm	19(95.0)			1(5.0)		
Differentiation						0.705
Well	3(100)			0(0)		
Moderate	25(80.6)			6(19.4)		
Poor	0(0)			0(0)		
Lymphovascular Invasion						**0.012**
Yes	18(81.8)			4(18.2)		
No	10(83.3)			2(16.7)		
Perineural Invasion						**0.005**
Yes	5(83.3)			1(16.7)		
No	23(82.1)			5(17.9)		
pT Staging						1.594
T_1_	1(100)			0(0)		
T_2_	9(90.0)			1(10.0)		
T_3_	16(76.2)			5(23.8)		
T_4_	2(100)			0(0)		
pN Staging						5.985
N_0_	11(78.6)			3(21.4)		
N_1_	13(100)			0(0)		
N_2_	4(57.1)			3(42.9)		
Pattern of Invasion						1.807
Pushing	13(92.9)			1(7.1)		
Infiltrative	15(75.0)			5(25.0)		
Intratumoral TILs						0.705
Mild	25(80.6)			6(19.4)		
Marked	3(100)			0(0)		
Peritumoral TILs						1.503
Mild	17(89.5)			2(10.5)		
Marked	11(73.3)			4(26.7)		
Tumor Budding						7.235
Yes	2(40.0)			3(60.0)		
No	26(89.7)			3(10.3)		
Mucin Pools						0.074
Yes	11(84.6)			2(15.4)		
No	17(81.0)			4(19.0)		

**Table 3:** Immunohistochemical tissue expression of PD-L1 in the tumor-infiltrating immune cells (TIICs) in the CRC patients

	^PD-L1 (TIICs)^				
	^Low^		^High^		^P-value^
^Parameter^	^N (%)^		^N (%)^		
^Age^					^3.848^
^≥60 Years^		^11(68.75)^		^5(31.25)^	
^<60 years^		^17(94.45)^		^1(5.55)^	
^Gender^					^0.672^
^Male^		^14(82.4)^		^3(17.6)^	
^Female^		^14(82.4)^		^3(17.6)^	
^Location^					^0.475^
^Right^		^12(85.72)^		^2(14.28)^	
^Left^		^15(78.94)^		^4(21.06)^	
^Right & Left^		^1(100)^		^0(0)^	
^Growth Type^					^1.807^
^Ulceroinfiltrative^		^13(92.85)^		^1(7.15)^	
^Ulceroproliferative^		^15(75)^		^5(25)^	
^Tumor Size^					^0.185^
^< 5cm^		^12(85.72)^		^2(14.28)^	
^≥ 5cm^		^16(80)^		^4(20)^	
^Differentiation^					^0.705^
^Well^		^3(100)^		^0(0)^	
^Moderate^		^25(80.64)^		^6(19.36)^	
^Poor^		^0(0)^		^0(0)^	
^Lymphovascular Invasion^					^7.362^
^Yes^		^21(95.45)^		^1(4.55)^	
^No^		^7(58.34)^		^5(41.66)^	
^Perineural Invasion^					^1.561^
^Yes^		^6(100)^		^0(0)^	
^No^		^22(78.58)^		^6(21.42)^	
^pT Staging^					^1.856^
^T1^		^1(100)^		^0(0)^	
^T2^		^7(70)^		^3(30)^	
^T3^		^18(85.72)^		^3(14.28)^	
^T4^		^2(100)^		^0(0)^	
^pN Staging^					^2.091^
^N0^		^10(71.42)^		^4(28.58)^	
^N1^		^12(92.30)^		^1(7.70)^	
^N2^		^6(85.71)^		^1(14.29)^	
^Pattern of Invasion^					^1.954^
^Pushing^		^10(71.42)^		^4(28.58)^	
^Infiltrative^		^18(90)^		^2(10)^	
^Intratumoral TILs^					^0.557^
^Mild^		^26(83.87)^		^5(16.13)^	
^Marked^		^2(66.66)^		^1(33.34)^	
^Peritumoral TILs^					^4.545^
^Mild^		^18(94.73)^		^1(5.27)^	
^Marked^		^10(66.66)^		^5(33.34)^	
^Tumor Budding^					^0.022^
^Yes^		^4(80)^		^1(20)^	
^No^		^24(82.76)^		^5(17.24)^	
^Mucin Pool(s)^					^1.435^
^Yes^		^12(92.30)^		^1(7.70)^	
^No^		^16(76.19)^		^5(23.81)^	

## Discussion

Colorectal adenocarcinoma is the most frequent gastrointestinal malignancy, causing morbidity and mortality around the world. It is more common in older people, with males being slightly more affected than females (1). 

In colorectal cancer, aggressive surgery combined with chemoradiotherapy is the backbone of treatment. Even though roughly 90% of patients with early-stage cancer who receive standardized treatment have a 5-year life expectancy, about 40% of them will still develop distant metastasis and/or local recurrence. This has prompted researchers to look into more effective treatments, such as immunotherapy (8). Amongst these, checkpoint blockade drugs that target PD-1 and its ligand, PD-L1, have attained unmatched therapeutic impact in various malignancies (5). The patients' average age and gender are concordant with a study by Shi SJ* et al. *and Shan T *et al.* (9, 10), and PD-L1 expression did not show any association with age or gender in most studies. Nevertheless, age has been described as an independent prognostic factor and also in association with comorbidities and ulcerative type of growth (11, 12).

Rosenbaum MW* et al. *and Masugi Y found a significant association of PD-L1 expression with gender, which could be an incidental finding (13, 14). In a meta-analysis of 13 retrospective cohort studies and one randomized controlled trial, patient gender was the only significant predictor of relative survival advantage. However, the reasons leading to this effect remain unclear. In a univariate regression study, women were found to have a greater recurrence-free and overall survival rate than men. It is hypothesized that female hormones may have a preventive effect against CRC (16).

In diverse studies, the IHC expression of PD-L1 on tumor cells ranges from 4.4% to 88.8%. The present study is consistent with studies by Zhu H* et al. *and Aziz ZW* et al. *(16, 17, 18, 19). These variations could be ascribed to scoring system differences, intratumoral staining heterogeneity due to tissue microarrays versus whole slides, positive cut-offs used, and other factors. 

To minimize the overestimation of PD-L1 expression and difficulty in the detection of very few positive neoplastic cells, PD-L1 expression was evaluated with a cut-off of >5%.

PD-L1 expression on TIICs is similar to the findings of Wang L* et al. *(20). Many studies have looked at the role of immune invading cells using specific markers such as PD-1 and CD8+. Higher PD-L1 expression on TIICs in some studies could be due to a variety of factors, including bigger sample sizes, a lack of specific criteria for PD-L1 positivity on the immune cells, and different cut-off values employed.

**Relationship Between PD-L1 Staining on the Tumoral Cells and Other Clinicopathological Features**

The expression of PD-L1 staining on the tumoral cells was not significant with the site and size of the tumor, which may be limited by the small sample size. However, a significant correlation of PD-L1 with both these features is noted by several authors. (5, 18, 21, 22). Many studies have analyzed the role of mutational status, such as KRAS, and BRAF, and key marker expression, such as HER2 and EGFR, on the impact of tumor site on prognosis. When compared to the right side, the left side was observed to be associated with a considerably longer progression-free survival and a superior overall survival in these trials (23).

In patients with colorectal adenocarcinoma of the infiltrative type, tumor size is found to be an independent determinant for overall survival and disease-free survival, but only for overall survival in patients with ulcerative type (12). 

There was no significant relationship between PD-L1 expression on the tumoral cells and tumor grade. Shi SJ* et al. *found a higher number of well-differentiated carcinomas in their analysis and a strong association with PD-L1 expression. They reasoned that PD-L1 may have an oncogenic role in colon cancer development by modulating cell proliferation, apoptosis, migration, invasion, and differentiation. There was a statistically significant difference in overall survival between the positive and negative groups. They also found that having high levels of PD-L1 increased the chance of mortality and decreased overall survival, implying that PD-L1 expression is an independent predictor of prognosis (9). 

A significant association of PD-L1 expression with LVI and PNI was noted. These results are comparable to those found by Droeser RA* et al. *and Huang CY* et al. *(19, 24). However, compared to their work, the sample size in our study is substantially smaller. PD-L1 expression is strongly linked with TNM stage, lymph node metastasis, and distant metastasis in a few studies, and it has also been established that elevated PD-L1 expression in tumor tissue is a poor predictive indicator on its own (10).

In many studies, the association between PD-L1 expression and T stage was insignificant, despite the sample size being substantial in a few of them. In their multivariate study, Shan T *et al.*, Masugi Y* et al., *and Droeser RA* et al. *found a significant correlation and found PD-L1 to be an independent risk factor (10, 14, 19). Enkhabat* et al. *investigated the link between PD-1, PD-L1, TGF-ß, and FOX-1 and discovered that PD-L1 positive groups had inferior overall survival rates. The present study had no association with tumor and nodal stage (18). 

In a multivariate analysis of 1363 cases, Shan T* et al. *and Droeser RA* et al. *discovered a substantial correlation between PD-L1 expression and the N stage. Poor tumor differentiation, lymph node metastases, and positive PD- L1 expression were all found to have an impact on prognosis in their study (10, 19). 

The tumor border is seen frequently in CRC cases with a low risk of distant and nodal metastasis and is associated with mismatch-repair deficiency, whereas an infiltrative tumor border configuration is associated with adverse clinicopathological features, early disease recurrence, poor survival, and molecular alterations related to aggressive tumor behavior, such as BRAFV600 mutation (25).

The epithelial-mesenchymal transition is thought to play a role in poor differentiation, infiltrating margin, and tumor budding. 

We did not find a meaningful correlation with margins, tumor budding, and extracellular mucin. Kim JH* et al. *(26) found an association between positive PD-L1 expression on tumor cells and poor differentiation, decreased extracellular mucin component, infiltrating growth pattern, tumor budding, LVI, and advanced stage in their study on MSI-H (microsatellite instability) CRC. In MMR (mismatch repair status)-competent CRC, Droeser RA* et al. *discovered a link between mucinous histology and PD-L1 expression (19). 

**Relationship Between PD-L1 on the Tumor-Infiltrating Immune Cells and Clinicopathological Features**

In 1992, Japanese researchers Ishida Y* et al. *identified and designated Programmed cell death protein 1 (PD-1) as an inhibitory checkpoint molecule produced on the surface of activated T-cells to regulate activation and proliferation (27). Activated T-cells, B-cells, macrophages, tumor cells, dendritic cells, and endothelial cells express PD-L1, a PD-1 ligand (28). In many malignancies, PD-L1 on immune cells may play a substantial role in the T-cell inhibitory mechanism (29, 30). Many researchers have looked at PD-1 and CD8+ immunostaining expression on invading immune cells. The use of PD-L1 in conjunction with them may be more relevant. 

This study had a significant connection between PD-L1 staining on the lymphocytes and tumor budding. These findings are comparable to those of Ahtiainen M *et al.*, who looked at the combined predictive value of PD-L1/PD-1 expression and immune cell infiltration in CRC as a function of MMR and discovered that combining the immune cell score of PD-1 and PD-L1 is a powerful independent prognostic factor for survival (7). The Crohn's-like disease lymphoid response of the host is associated with a lower incidence of lymph node metastases and improved survival in a few studies (31, 32). 

Shi S J *et al.* found a link between high PD-L1 expression and higher tumor-related mortality in CRC, implying that it could be used as a biomarker for poor prognosis (9). 

Droeser* et al., *on the other hand, found that high PD-L1 expression in colorectal cancer was linked to a low tumor grade, early T stage, lack of vascular invasion and lymph node metastases, and enhanced patient survival. They discovered the aforementioned finding in MMR proficient CRC because this study was a multivariate analysis for numerous clinicopathological variables with MMR status. They hypothesized that the link between PD-L1 expression in CRC cells and a better prognosis in MMR-positive CRC was due to increased CD8+ T cell infiltration (19). 

Shen Z* et al. *concluded in a comprehensive review and meta-analysis that PD-L1 expression might be used as an independent factor for predicting CRC prognosis because patients with advanced disease or lymphatic invasion are more likely to express PD-L1 (33). 

PD-L1 expression in the tumoral cells was substantially related to poor right colon cancer, poor differentiation, and poor overall survival in another meta-analysis by Li Y *et al.*, which included ten papers (34).

In a meta-analysis involving 32 papers and 8823 CRC patients, Wang S* et al. *found that PD-L1 expression was associated with lymphatic metastasis, tumor diameter, differentiation, and vascular invasion and thus concluded that PD-L1 expression is an independent predictor of poor prognosis in CRC (35). 

Although PD-L1 expression on tumor cells indicates a tumor's chance of responding to anti-PD-L1 therapy, its assessment is also an important biomarker for determining prognosis in CRC.

## Conclusion

CRC is one of the most frequent tumors in the Indian subcontinent and is an aggressive malignancy. Currently, the basic treatment for this condition is radical surgery combined with chemoradiotherapy. In the current study, the immunohistochemical expression of PD-L1 on the tumoral cells and in the tumor microenvironment in CRC was found to be associated with LVI, PNI, and tumor budding, all of which are recognized as poor prognostic factors. There is, however, no correlation with other known prognostic variables. Various studies have found contradictory outcomes with numerous clinicopathological variables. A small sample size limits this study. Studies with larger samples may be required to confirm the role of PD-L1 as an independent predictive factor. Expression of PD-1 and CD8+ on lymphocytes would have refined this study. Molecular phenotyping of the CRC was impossible, and recurrence or survival analysis was not addressed as the follow-up data was unavailable. Since anti-PD-L1 may be used for targeted therapy, evaluating the immunohistochemical expression of PD-L1 in CRC in a country like ours may be beneficial.

## Ethics Approval & Consent to Participate

Ethics approval was obtained from the Institutional Ethics Committee, and all the participants filled out consent forms. 

## Authors' Contributions

SCS, MS: Study conception and design; ST, SCS: IHC analysis and interpretation; ST: Data collection; SCS, MS: Initial manuscript drafting; SCS, MS: Revised it critically for important intellectual content.

## Conflict of Interest

The authors declared no conflict of interest.

## References

[1] GLOBOCAN 2020: New Global Cancer Data [Internet].

[2] Sharma D, Singh G (2017). Clinico-pathological profile of colorectal cancer in first two decades of life: A retrospective analysis from tertiary health center. Indian J Cancer.

[3] Nagtegaal ID, Arends MJ, Salto-Tellez M (2019). WHO Classification of Tumors: Digestive System Tumors.

[4] Wang HB, Yao H, Li CS, Liang LX, Zhang Y, Chen YX (2017). Rise of PD-L1 expression during metastasis of colorectal cancer: Implications for immunotherapy. J Dig Dis.

[5] Lee LH, Cavalcanti MS, Segal NH, Hechtman JF, Weiser MR, Smith JJ (2016). Patterns and prognostic relevance of PD-1 and PD-L1 expression in colorectal carcinoma. Mod Pathol.

[6] Amin MB, Edge SB (2017). AJCC cancer staging manual.

[7] Ahtiainen M, Wirta EV, Kuopio T, Seppälä T, Rantala J, Mecklin JP (2019). Combined prognostic value of CD274 (PD-L1)/PDCDI (PD-1) expression and immune cell infiltration in colorectal cancer as per mismatch repair status. Mod Pathol.

[8] Wu Z, Yang L, Shi L, Song H, Shi P, Yang T (2019). Prognostic Impact of Adenosine Receptor 2 (A2aR) and Programmed Cell Death Ligand 1 (PD-L1) Expression in Colorectal Cancer. Biomed Res Int.

[9] Wang LJ, Wang GD, Guo ZY, Wei M, Meng YL (2013). B7-H1 expression is associated with poor prognosis in colorectal carcinoma and regulates the proliferation and invasion of HCT116 colorectal cancer cells. PLoS One.

[10] Yamano T, Yamauchi S, Kimura K, Babaya A, Hamanaka M, Kobayashi M (2017). Influence of age and comorbidity on prognosis and application of adjuvant chemotherapy in elderly Japanese patients with colorectal cancer: A retrospective multicentre study. Eur J Cancer..

[11] Dai W, Li Y, Meng X, Cai S, Li Q, Cai G (2017). Does tumor size have its prognostic role in colorectal cancer? Re-evaluating its value in colorectal adenocarcinoma with different macroscopic growth pattern. Int J Surg..

[12] Shan T, Chen S, Wu T, Yang Y, Li S, Chen X (2019). PD-L1 expression in colon cancer and its relationship with clinical prognosis. Int J Clin Exp Pathol.

[13] Rosenbaum MW, Bledsoe JR, Morales-Oyarvide V, Huynh TG, Mino-Kenudson M (2016). PD-L1 expression in colorectal cancer is associated with microsatellite instability, BRAF mutation, medullary morphology and cytotoxic tumor-infiltrating lymphocytes. Mod Pathol.

[14] Masugi Y, Nishihara R, Yang J, Mima K, da Silva A, Shi Y (2017 ). Tumor CD274 (PD-L1) expression and T cells in colorectal cancer. Gut.

[15] Schmuck R, Gerken M, Teegen EM, Krebs I, Klinkhammer-Schalke M, Aigner F (2020). Gender comparison of clinical, histopathological, therapeutic and outcome factors in 185,967 colon cancer patients. Langenbecks Arch Surg.

[16] Zhu H, Qin H, Huang Z, Li S, Zhu X, He J (2015). Clinical significance of programmed death ligand-1 (PD-L1) in colorectal serrated adenocarcinoma. Int J Clin Exp Pathol.

[17] Al-hayali ZW, Mahmood AM, Yahiya ZO, Taib Al--Nuaimy WM (2020). Correlation between programmed cell death ligand1 (PD-L1) expression and clinical parameters in colorectal carcinoma. J Contem Med Sci.

[18] Enkhbat T, Nishi M, Takasu C, Yoshikawa K, Jun H, Tokunaga T et al (2018). Programmed Cell Death Ligand 1 Expression Is an Independent Prognostic Factor in Colorectal Cancer. Anticancer Res.

[19] Droeser RA, Hirt C, Viehl CT, Frey DM, Nebiker C, Huber X (2013). Clinical impact of programmed cell death ligand 1 expression in colorectal cancer. Eur J Cancer.

[20] Wang L, Ren F, Wang Q, Baldridge LA, Monn MF, Fisher KW (2016). Significance of Programmed Death Ligand 1 (PD-L1) Immunohistochemical Expression in ColorectalCancer. Mol Diagn Ther.

[21] Lee KS, Kim BH, Oh HK, Kim DW, Kang SB, Kim H (2018). Programmed cell death ligand-1 protein expression and CD274/PD-L1 gene amplification in colorectal cancer: Implications for prognosis. Cancer Sci.

[22] Stintzing S, Tejpar S, Gibbs P, Thiebach L, Lenz HJ (2017). Understanding the role of primary tumor localisation in colorectal cancer treatment and outcomes. Eur J Cancer..

[23] Jiang H, Zhang R, Jiang H, Zhang M, Guo W, Zhang J (2020). Retrospective analysis of the prognostic value of PD-L1 expression and 18F-FDG PET/CT metabolic parameters in colorectal cancer. J Cancer.

[24] Huang CY, Chiang SF, Ke TW, Chen TW, You YS, Chen WT (2018). Clinical significance of programmed death 1 ligand-1 (CD274/PD-L1) and intra-tumoral CD8+ T-cell infiltration in stage II-III colorectal cancer. Sci Rep.

[25] Koelzer VH, Lugli A (2014). The tumor border configuration of colorectal cancer as a histomorphological prognostic indicator. Front Oncol..

[26] Kim JH, Park HE, Cho NY, Lee HS, Kang GH (2016). Characterisation of PD-L1-positive subsets of microsatellite-unstable colorectal cancers. Br J Cancer.

[27] Ishida Y, Agata Y, Shibahara K, Honjo T (1992). Induced expression of PD-1, a novel member of the immunoglobulin gene superfamily, upon programmed cell death. EMBO J.

[28] Hansen JD, Du Pasquier L, Lefranc MP, Lopez V, Benmansour A, Boudinot P (2009). The B7 family of immunoregulatory receptors: a comparative and evolutionary perspective. Mol Immunol.

[29] Dong H, Zhu G, Tamada K, Chen L (1999). B7-H1, a third member of the B7 family, co-stimulates T-cell proliferation and interleukin-10 secretion. Nat Med.

[30] Carter L, Fouser LA, Jussif J, Fitz L, Deng B, Wood CR (2002). PD-1:PD-L inhibitory pathway affects both CD4(+) and CD8(+) T cells and is overcome by IL-2. Eur J Immunol.

[31] Washington MK (2008). Colorectal carcinoma: selected issues in pathologic examination and staging and determination of prognostic factors. Arch Pathol Lab Med.

[32] Ogino S, Nosho K, Irahara N, Meyerhardt JA, Baba Y, Shima K (2009). Lymphocytic reaction to colorectal cancer is associated with longer survival, independent of lymph node count, microsatellite instability, and CpG island methylator phenotype. Clin Cancer Res.

[33] Shen Z, Gu L, Mao D, Chen M, Jin R (2019). Clinicopathological and prognostic significance of PD-L1 expression in colorectal cancer: a systematic review and meta-analysis. World J Surg Oncol.

[34] Li Y, He M, Zhou Y, Yang C, Wei S, Bian X (2019). The Prognostic and Clinicopathological Roles of PD-L1 Expression in Colorectal Cancer: A Systematic Review and Meta-Analysis. Front Pharmacol..

[35] Wang S, Yuan B, Wang Y, Li M, Liu X, Cao J (2021). Clinicopathological and prognostic significance of PD-L1 expression in colorectal cancer: a meta-analysis. Int J Colorectal Dis.

